# RGS6 Drives Spinal Cord Injury by Inhibiting AMPK Pathway in Mice

**DOI:** 10.1155/2022/4535652

**Published:** 2022-04-25

**Authors:** Wenxin Dao, Zhe Xiao, Weize Yang, Xiaomin Luo, Hongxia Xia, Zuneng Lu

**Affiliations:** Department of Neurology, Renmin Hospital of Wuhan University, Wuhan, 430060 Hubei, China

## Abstract

**Objective:**

Oxidative stress and inflammation play critical roles in the pathogenesis of spinal cord injury (SCI). Regulator of G protein signaling 6 (RGS6) is involved in controlling ROS generation and inflammatory response under different contexts. This study is aimed at investigating its role and underlying mechanism in SCI.

**Methods:**

Contusive SCI mouse models were generated, and lentiviral vectors were injected to silence or overexpress RGS6 in the spinal cord. To inhibit AMP-activated protein kinase (AMPK) activity, SCI mice were intraperitoneally injected with compound C (20 mg/kg) every two days. Oxidative and inflammatory markers were detected.

**Results:**

Spinal RGS6 expression was elevated upon SCI stimulation. RGS6 knockdown suppressed, while RGS6 overexpression aggravated oxidative stress, inflammation, and SCI in mice. Mechanistically, RGS6 elevation during SCI deactivated AMPK pathway, thereby exacerbating oxidative stress and inflammation in SCI mice.

**Conclusion:**

RGS6 is required for the initiation and progression of SCI, and knocking down RGS6 may provide promising therapeutic strategies for SCI patients.

## 1. Introduction

Spinal cord injury (SCI) is a devastating neurological damage that eventually leads to sensory, motor, and autonomic dysfunction. Despite the improvement of surgeries, medications, and rehabilitations, the prognosis of SCI remains unsatisfactory. Oxidative stress and inflammation have been verified to play critical roles in the pathogenesis of SCI, and inhibiting reactive oxygen species (ROS) overproduction and inflammation significantly promotes functional recovery after SCI [1, 2]. AMP-activated protein kinase (AMPK) is a serine/threonine protein kinase and regulates numerous biological processes, including oxidative stress and inflammation [3, 4]. Hu et al. revealed that AMPK activation upregulates uncoupling protein 2 level and subsequently suppresses doxorubicin-induced ROS overproduction in the myocardium [5]. AMPK activation also inhibits oxidative damage and inflammatory response in SCI mice, thereby promoting the recovery of spinal function [6, 7]. These findings identify AMPK as a promising therapeutic target of SCI.

Regulator of G protein signaling (RGS) proteins have emerged as critical modulators of intracellular signaling axis, modulating multiple biological functions. RGS6 belongs to the R7 subfamily of RGS proteins and is involved in controlling ROS generation as well as inflammatory response under different contexts. Maity et al. revealed that RGS6 induced mitochondrial membrane potential loss, ROS accumulation, and intrinsic apoptotic pathways and that RGS6 deletion in mouse embryonic fibroblasts significantly compromised doxorubicin-induced apoptosis [8]. A very recent study demonstrated that hepatic RGS6 increased ROS generation and inflammation, thereby driving lipid deposition, cell death, and nonalcoholic fatty liver disease [9]. RGS6 is widely expressed in the nervous system and contributes to neuronal differentiation, parasympathetic activation, and neurodegeneration [10–12]. In general, RGS6 is a novel therapeutic target in central nervous system diseases; however, its role and underlying mechanism in SCI remain elusive. The present study demonstrated that RGS6 induced oxidative stress and inflammation in SCI mice and that RGS6 knockdown prevented SCI by activating AMPK pathway.

## 2. Materials and Methods

### 2.1. Reagents

Compound C (CC, S7306) was purchased from Selleck Chemicals (Houston, TX, USA). Pierce™ ECL Western Blotting Substrate (32106) and 2′,7′-dichlorodihydrofluorescein diacetate (DCFH-DA, D399) were purchased from Invitrogen (Carlsbad, CA, USA). Colorimetric lipid peroxidation assay kit (ab118970), total antioxidant capacity (TAOC) assay kit (ab65329), mouse interleukin-1 beta (IL-1*β*, ab197742), IL-6 (ab222503), IL-18 (ab216165), and tumor necrosis factor-alpha (TNF-*α*, ab208348) ELISA kits, and fluorometric caspase-1 assay kit (ab39412) were purchased from Abcam (Cambridge, MA, USA). Lentiviral vectors carrying short hairpin RNA against mouse RGS6 (sh*Rgs6*), scramble sh*Ctrl*, full-length RGS6 (*Rgs6*), or scramble *Ctrl* were generated by Shanghai GeneChem Co., Ltd. (Shanghai, China).

### 2.2. Contusive SCI Surgery and Animal Treatments

Contusive SCI surgery was performed in 5% isoflurane-anesthetized mice as previously described [13]. Briefly, mice received a laminectomy at T8 to T10 of spinal cords on a surgical microscope, and then, the spinal cord was contused at T9 by a weight (10 g) from 5 cm height using a mouse spinal cord impactor. The success of this model was validated by body trembling, dropped tails, and a fluttering retraction of the hind limbs and body. In sham-operated mice, the skin and muscle were separated, but no SCI was introduced. After surgery, mice were disinfected with povidone-iodine solution and rehydrated with 0.9% sterile saline, and then, their bladders were manually evacuated twice daily until the restoration of urinating function. To knock down or overexpress RGS6 in the spinal cord, mice were intraspinally injected with 2 *μ*L lentivirus carrying sh*Rgs6* or *Rgs6* at rostral and caudal sites 1 mm from the lesion epicenter immediately post-SCI (~0.5 mm in depth) [14]. To inhibit AMPK activity, SCI mice were intraperitoneally injected with CC (20 mg/kg) every two days beginning at one week pre-SCI [15]. Seven days after SCI surgery, these mice were sacrificed for molecular detection.

### 2.3. Tactile and Thermal Sensation Tests

Tactile sensation was measured using Von Frey filament test as previously described [16]. Briefly, the hind paw was stimulated with a monofilament, and the positive response was identified as rapid hind paw withdrawal or sudden licking. The withdrawal threshold force producing more than 50% positive responses was used to calculate tactile sensation at 7 days post-SCI. Thermal sensation was evaluated by the unilateral Hargreaves thermal test [16]. After acclimating for 1 h in the testing environment, mouse hind paw was stimulated with infrared radiation, and thermal sensation was monitored using a fiber-optic sensor on the movable infrared heat source. Rapid withdrawal or sudden licking of hind paw was identified as the positive response.

### 2.4. Evaluation of Blood-Spinal Cord Barrier Permeability

Blood-spinal cord barrier permeability was determined by measuring extravasation of Evans blue from the spinal cord lesion as previously described [17]. Briefly, mice were intravenously injected with 2% Evans blue dye at 7 days post-SCI and then allowed circulating for 3 h. Next, the lesion was homogenized in 50% trichloroacetic acid solution, incubated at 60°C for 24 h and centrifuged at 10 000 g for 10 min, and then, the supernatants were collected and measured at the excitation/emission wavelength of 620/680 nm.

### 2.5. Basso Mouse Scale (BMS) Behavioral Analysis

BMS behavioral analysis was performed to determine locomotive function of hind limbs in SCI mice according to the hind-limb joint activities, front and rear limbs coordination, trunk position and stability, paw posture, toe clearance, and tail posture scoring system [13, 17]. All mice were blindly evaluated by two independent observers and scored from 0 to 9 points.

### 2.6. Western Blot

Western blot was performed according to standard protocols [18, 19]. Briefly, spinal cords were homogenized in the RIPA lysis buffer, centrifuged at 12 000 rpm for 30 min, quantified by BCA method, mixed with 5× loading buffer and boiled at 100°C for 10 min. Next, 20 *μ*g total proteins were separated by 10% SDS-PAGE and transferred onto PVDF membranes. After being blocked by 5% skim milk, membranes were incubated with the following primary antibodies at 4°C overnight. Anti-RGS6 (sc-271643; Santa Cruz Biotechnology, Dallas, Texas, USA), antiglyceraldehyde-3-phosphate dehydrogenase (GAPDH, 5174; Cell Signaling Technology, Danvers, MA, USA), antithioredoxin interacting protein (TXNIP, ab188865; Abcam), antinucleotide-binding domain-like receptor protein 3 (NLRP3, ab263899; Abcam), antiphospho-AMPK (Thr172, p-AMPK, 50081; Cell Signaling Technology), antitotal AMPK (t-AMPK, 5831; Cell Signaling Technology). On the second day, membranes were incubated with horseradish peroxidase-conjugated secondary antibodies, and detected by the Pierce™ ECL Western Blotting Substrate.

### 2.7. Quantitative Real-Time PCR

Total RNA was extracted from the spinal cord using TRIzol (Invitrogen), reversely transcribed to cDNA using the PrimeScript RT reagent Kit (Takara, Shiga, Japan) and then exposed to quantitative real-time PCR analysis using the SYBR Premix Ex Taq (Takara). Relative mRNA expressions were calculated using the 2^−△△Ct^ method, and GAPDH was used as an internal control [20, 21].

### 2.8. Detection of Oxidative Stress

The nonfluorescent DCFH-DA can be cleaved and oxidized to fluorescent DCF by excessive free radicals, so it was used to evaluate ROS level in the spinal cord as previously described [22, 23]. Briefly, fresh spinal cords were homogenized and incubated with DCFH-DA (50 *μ*mol/L) at 37°C for 30 min, and then, DCF intensities were measured at the excitation/emission wavelength of 485/535 nm. Malondialdehyde (MDA) is a product of lipid peroxidation, and spinal MDA level was determined using a colorimetric lipid peroxidation assay kit according to the manufacturer's instructions. Endogenous antioxidant capacity was determined using a commercial kit. In brief, fresh spinal cords were homogenized and centrifuged, and then, the supernatants were incubated with 100 *μ*L Cu^2+^ Working Solution at room temperature for 90 min and measured at 570 nm.

### 2.9. Biochemical Analysis

Spinal IL-1*β*, IL-6, IL-18, and TNF-*α* levels were detected using commercial ELISA kits according to the manufacturer's instructions. Caspase-1 activity in the spinal cord was determined by detecting the cleavage of YVAD-AFC. Briefly, fresh spinal cords were homogenized and centrifuged, and then, the supernatants were incubated with YVAD-AFC (1 mmol/L) at 37°C for 2 h and measured at the excitation/emission wavelength of 400/505 nm.

### 2.10. Statistical Analysis

All data were presented in the form of means ± S.D. and analyzed using SPSS software (Version 19.0). Unpaired Student's *t* test was used to compare differences between 2 groups, and one-way ANOVA followed by Tukey's post hoc test was performed for the analysis of the differences among 3 or more groups. *P* < 0.05 was considered statistically significant.

## 3. Results

### 3.1. RGS6 Knockdown Prevents SCI in Mice

**Figure 1:**
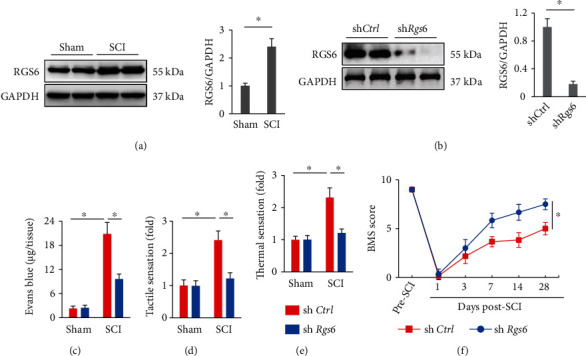
RGS6 knockdown prevents SCI in mice. (a) RGS6 protein levels were examined by Western blot at 7 days post-SCI. (b) The efficiency of sh*Rgs6* in mice. (c) Quantification of Evans blue content in the spinal cord at 7 days post-SCI. (d, e) Sensitivities to tactile and thermal stimulation at 7 days post-SCI. (f) BMS scoring to evaluate locomotive function at indicated time pre- or post-SCI. Bars indicate means ± S.D., *n* = 6 per group. ^∗^*P* < 0.05 compared with indicated groups.

To explore the potential involvement of RGS6 in SCI development, we first evaluated whether spinal RGS6 expression was altered upon SCI stimulation. Western blot results indicated that RGS6 protein levels were dramatically increased in the spinal cord of SCI mice (Figure 1(a)). To investigate whether elevated RGS6 expression contributes to SCI in mice, sh*Rgs6* was intraspinally injected to knock down endogenous RGS6 (Figure 1(b)). It is well-accepted that blood-spinal cord barrier is disrupted upon SCI. As shown in Figure 1(c), Evans blue extravasation to the spinal cord was increased in SCI mice, but dramatically decreased by RGS6 knockdown. RGS6 silence also decreased the withdraw thresholds responding to mechanical stimuli and heat stimuli, indicating an improvement of hyperesthesia in *Rgs6*-suppressed mice (Figures 1(d) and 1(e)). In addition, the BMS scores of sh*Rgs6*-injected mice were significantly higher than those injected with sh*Ctrl* post-SCI, indicating the improved locomotive function (Figure 1(f)). These findings demonstrate that RGS6 knockdown prevents SCI in mice.

### 3.2. RGS6 Knockdown Suppresses Oxidative Stress and Inflammation in SCI Mice

**Figure 2:**
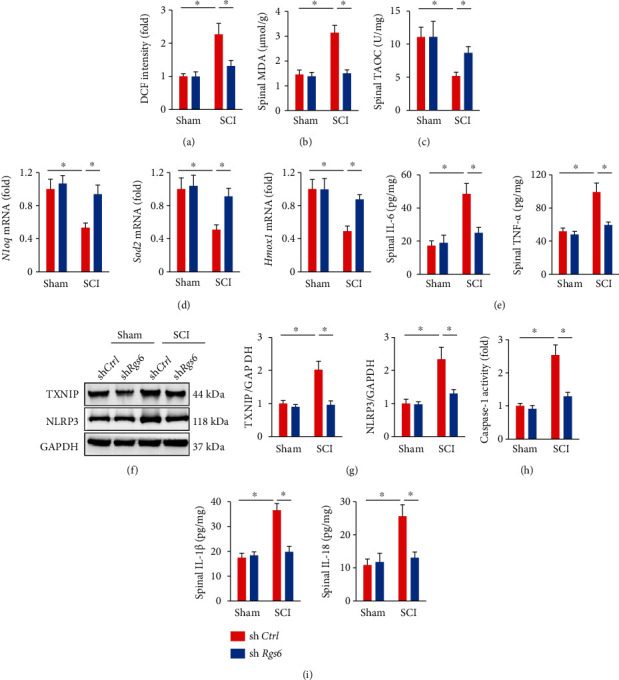
RGS6 knockdown suppresses oxidative stress and inflammation in SCI mice. (a) ROS levels in the spinal cord at 7 days post-SCI were assessed by DCF intensity. (b, c) Spinal MDA and TAOC levels at 7 days post-SCI. (d) Relative mRNA levels of *Nqo1*, *Sod2*, and *Hmox1* in the spinal cord. (e) Spinal IL-6 and TNF-*α* levels. (f, g) TXNIP and NLRP3 protein levels were examined by Western blot at 7 days post-SCI. (h) Relative caspase-1 activity in the spinal cord at 7 days post-SCI. (i) Spinal IL-1*β* and IL-18 levels. Bars indicate means ± S.D., *n* = 6 per group. ^∗^*P* < 0.05 compared with indicated groups.

ROS overproduction is a critical pathogenic factor of SCI and leads to oxidative damage to cellular components [7]. We detected excessive accumulations of free radicals in the spinal cord from SCI mice, accompanied by an increased MDA level, which were dramatically suppressed by RGS6 knockdown (Figures 2(a) and 2(b)). Accordingly, the decreased spinal TAOC in SCI mice was elevated by sh*Rgs6* injection (Figure 2(c)). Meanwhile, RGS6 silence also restored the expression of antioxidant molecules in SCI mice, as evidenced by increased mRNA levels of NAD(P)H quinone dehydrogenase 1 (*Nqo1*), superoxide dismutase 2 (*Sod2*), and heme oxygenase 1 (*Hmox1*) (Figure 2(d)). Inflammation is the other feature and contributor of SCI. As shown in Figure 2(e), spinal IL-6 and TNF-*α* levels were significantly increased upon SCI stimulation, but decreased in those injected with sh*Rgs6*. Upon oxidative stress, TXNIP detaches from thioredoxin, interacts with NLRP3, and then, activates NLRP3 inflammasome. Activation of NLRP3 inflammasome leads to the maturation and release of multiple proinflammatory cytokines (e.g., IL-1*β* and IL-18), which in turn aggravate inflammatory response and SCI [24]. As shown in Figures 2(f) and 2(g), TXNIP and NLRP3 protein levels were increased in the spinal cord from SCI mice, but decreased by sh*Rgs6* injection. Increased caspase-1 activity is a biomarker for the activation of NLRP3 inflammasome and contributes to the processing of proinflammatory cytokines. We found that RGS6 silence dramatically suppressed spinal caspase-1 activity in SCI mice (Figure 2(h)). Accordingly, spinal IL-1*β* and IL-18 levels were also reduced in those treated with sh*Rgs6* (Figure 2(i)). Collectively, we demonstrate that RGS6 knockdown suppresses oxidative stress and inflammation in SCI mice.

### 3.3. RGS6 Overexpression Aggravates Oxidative Stress, Inflammation, and SCI in Mice

**Figure 3:**
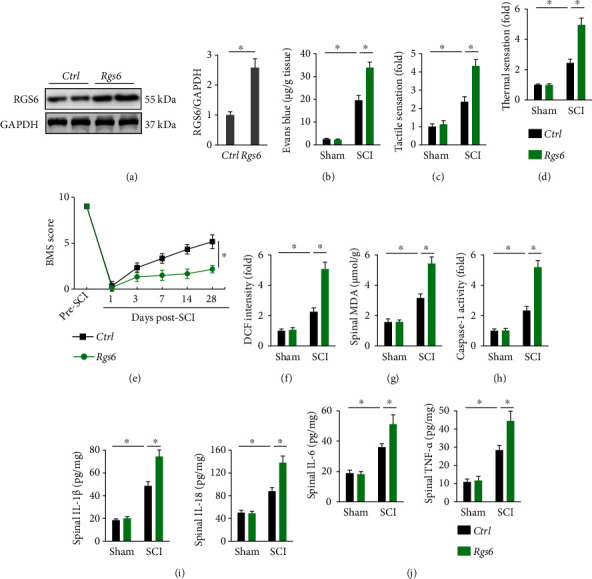
RGS6 overexpression aggravates oxidative stress, inflammation, and SCI in mice. (a) The efficiency of *Rgs6* in mice. (b) Quantification of Evans blue content in the spinal cord at 7 days post-SCI. (c, d) Sensitivities to tactile and thermal stimulation at 7 days post-SCI. (e) BMS scoring to evaluate locomotive function at indicated time pre- or post-SCI. (f) ROS levels in the spinal cord at 7 days post-SCI were assessed by DCF intensity. (g) Spinal MDA level at 7 days post-SCI. (h) Relative caspase-1 activity in the spinal cord at 7 days post-SCI. (i, j) Spinal IL-1*β*, IL-6, IL-18, and TNF-*α* levels. Bars indicate means ± S.D., *n* = 6 per group. ^∗^*P* < 0.05 compared with indicated groups.

Then, we overexpressed RGS6 in the spinal cord by injecting lentivirus carrying full-length *Rgs6* to investigate whether overexpressing RGS6 could aggravate SCI in mice (Figure 3(a)). As shown in Figure 3(b), RGS6 overexpression further disrupted the integrity of blood-spinal cord barrier in SCI mice, as evidenced by increased extravasation of Evans blue dye. In addition, the sensory and locomotive dysfunction were further exacerbated by RGS6 overexpression upon SCI stimulation (Figures 3(c)–3(e)). In line with the functional impairment, *Rgs6*-overexpressed SCI mice displayed increased ROS generation and lipid peroxidation (Figures 3(f) and 3(g)). Activation of NLRP3 inflammasome in SCI mice was also amplified in those with RGS6 overexpression, as evidenced by increased caspase-1 activity and spinal IL-1*β* and IL-18 levels (Figures 3(h) and 3(i)). In addition, *Rgs6*-overexpressed mice displayed elevated spinal IL-6 and TNF-*α* levels upon SCI stimulation (Figure 3(j)). Together, these data reveal that RGS6 overexpression aggravates oxidative stress, inflammation, and SCI in mice.

### 3.4. RGS6 Silence Alleviates SCI by Activating AMPK

**Figure 4:**
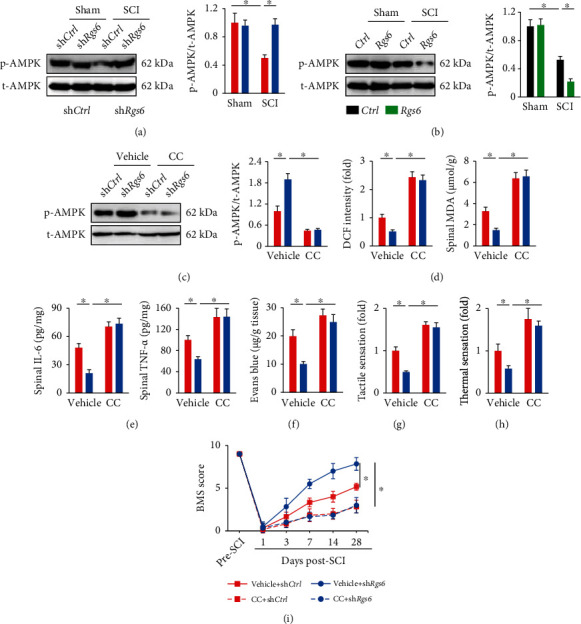
RGS6 silence alleviates SCI by activating AMPK. (a, b) AMPK phosphorylation in the spinal cord from *Rgs6*-silenced or overexpressed mice. (c) The inhibitory efficiency of CC on AMPK in mice. (d) ROS and MDA levels in the spinal cord at 7 days post-SCI were assessed by DCF intensity. (e) Spinal IL-6 and TNF-*α* levels. (f) Quantification of Evans blue content in the spinal cord at 7 days post-SCI. (g, h) Sensitivities to tactile and thermal stimulation at 7 days post-SCI. (i) BMS scoring to evaluate locomotive function at indicated time pre- or post-SCI. Bars indicate means ± S.D., *n* = 6 per group. ^∗^*P* < 0.05 compared with indicated groups.

AMPK is a multifunctional kinase and plays critical roles in regulating oxidative stress, inflammation, and SCI. In addition, our previous study indicated that AMPK activation prevented nerve injury upon ischemic stimulation [25]. Therefore, we tried to find out whether RGS6 silence alleviated SCI by activating AMPK. As shown in Figures 4(a) and 4(b), RGS6 silence restored, while RGS6 overexpression further reduced AMPK activation in SCI mice. To validate the causative role of AMPK activation in this process, SCI mice were injected with CC to inhibit endogenous AMPK activity. As shown in Figure 4(c), CC injection blocked AMPK activation in sh*Rgs6*-injected mice upon SCI stimulation. Accordingly, the antioxidant and anti-inflammatory effects of sh*Rgs6* were completely abolished in the presence of CC, as evidenced by increased DCF intensity, spinal MDA, IL-6, and TNF-*α* levels (Figures 4(d) and 4(e)). AMPK inhibition by CC also abolished the protection of blood-spinal cord barrier by sh*Rgs6* (Figure 4(f)). In addition, the improved sensory and locomotive functions in sh*Rgs6*-injected mice were blunted in those treated with CC (Figures 4(g)–4(i)). Taken together, we conclude that RGS6 silence alleviates SCI by activating AMPK.

## 4. Discussion

SCI is a severe traumatic event that causes sensory and motor dysfunction. The global incidence of traumatic SCI is 105 cases per million persons, and there are an estimated 768 473 new cases annually worldwide [26]. Despite the improvement of comprehensive treatment, the clinic outcome is still unsatisfactory. Therefore, it is urgently needed to elucidate the molecular basis of SCI progression and develop novel targeted therapies. In the present study, we found that spinal RGS6 expression was elevated upon SCI stimulation and that RGS6 knockdown dramatically prevented SCI in mice. In contrast, RGS6 overexpression further aggravated SCI. Mechanistically, RGS6 elevation during SCI deactivated AMPK pathway, thereby exacerbating oxidative stress and inflammation in SCI mice. In general, RGS6 is required for the initiation and progression of SCI, and knocking down RGS6 may provide promising therapeutic strategies for SCI patients.

Blood-spinal cord barrier is important for controlling spinal cord homeostasis; however, its functional and molecular integrities were dramatically disrupted immediately post-SCI, usually occurring within 5 min and lasting up to 28 days [27]. Disruption of blood-spinal cord barrier contributes to the extravasations of fluid and leukocytes, resulting in tissue edema and inflammatory response. Accordingly, we also observed a significant disruption of blood-spinal cord barrier in SCI mice, as evidenced by increased extravasation of Evans blue dye. This elevated permeability also potentiates the infiltration of circulating leukocytes to the spinal cord, eventually orchestrating a proinflammatory microenvironment in the spinal cord. The recruited leukocytes produce excessive free radicals and induce oxidative damage to cellular components. Meanwhile, endogenous antioxidant capacity is compromised by SCI stimulation, which further amplifies tissue injury. Upon oxidative stress, TXNIP binds to NLRP3 and activates NLRP3 inflammasome, releasing large amounts of mature IL-1*β* and IL-18. Accordingly, Jiao et al. previously revealed that selective inhibition of NLRP3 inflammasome dramatically suppressed spinal inflammation and improved neurological function in SCI mice [28]. In this study, we observed a significant activation of NLRP3 inflammasome in the spinal cord from SCI mice, which was suppressed by RGS6 silence.

RGS6, a member of RGS proteins, is first discovered in the nervous system and well-known for its role in regulating neuronal processes. Alike other RGS members, RGS6 is required for the pronounced acceleration of G*α* GTPase activity and plays critical roles in controlling classical G protein-coupled receptors (GPCR) pathways [12, 29]. AMPK is a nodal downstream effector of GPCR pathways and mediates multiple biological functions, including antioxidant and anti-inflammatory capacities. Wu et al. and Xu et al. independently demonstrated that AMPK activation dramatically augmented autophagy and inhibited pyroptosis, thereby preventing SCI in mice [30, 31]. In addition, AMPK activation also attenuated neuroinflammation and oxidative damage in SCI mice and ultimately promoted functional recovery [7, 32]. In this study, we showed that RGS6 silence activated, while RGS6 overexpression inhibited AMPK pathway in the spinal cord from SCI mice. Furthermore, AMPK inhibitor completely abolished the protective effects of sh*Rgs6* against SCI-induced oxidative stress and inflammation. Of note, there are some limitations of the present study. First, the primary cellular type expressing RGS6 in the spinal cord remains unknown. Second, whether RGS6 drives oxidative stress and inflammation in the spinal cord cells in vitro should be validated. Third, the specific mechanism by which RGS6 deactivates AMPK has not been determined in this study.

In summary, our findings firstly identify RGS6 as a positive regulator of SCI progression through promoting oxidative stress and inflammation, which is largely dependent on the suppression of AMPK pathway. These results suggest that RGS6 could be a promising therapeutic candidate to treat SCI.

## Data Availability

The data that support the findings of this study are available from the corresponding author upon reasonable request.
